# Significance of tumor deposits combined with lymph node metastasis in stage III colorectal cancer patients: a retrospective multi-center cohort study from China

**DOI:** 10.1007/s00384-022-04149-z

**Published:** 2022-05-20

**Authors:** Hongjiang Pu, Xiaolin Pang, Jiangping Fu, Rui Zheng, Yaxue Chen, Dafu Zhang, Xiangdong Fang

**Affiliations:** 1 Department of Radiology, The Third Affiliated Hospital of Kunming Medical University, Yunnan Cancer Hospital, Yunnan Cancer Centre, Kunming, 650118 Yunnan China; 2 Department of Oncology, Dazhou Central Hospital, Dazhou, 635000 Sichuan China; 3 Department of Radiotherapy, The Sixth Affiliated Hospital of Sun Yat-Sen University, Guangzhou, 510655 China; 4 Department of Nursing, Dazhou Vocational and Technical College, Dazhou, 635000 Sichuan China

**Keywords:** Colorectal cancer, Tumor deposits, Postoperative prognosis, Stage III

## Abstract

**Purpose:**

The study aimed to explore the value of tumor deposits in stage III colorectal cancer (CRC) and verify whether patients with more tumor deposit numbers have higher risk of recurrence.

**Methods:**

The retrospective cohort analysis was performed at two cancer centers of China. Stage III CRC patients who underwent radical resection at the center between April 2008 and February 2019 were identified. The Univariate/Multivariate Cox regression, Kaplan–Meier analysis, and PSM were recurrence-free survival (RFS) used.

**Results:**

Total 1080 stage III CRC patients (634 [58.7%] men; median [IQR] age, 60 [50–68] years) who underwent radical surgical resection were identified for inclusion in this study. Patients with tumor deposits had a 12.8% lower 3-year RFS (*n* = 236 [69.9%]) than the patients without tumor deposits (*n* = 844 [82.7%]) (*P* ≤ 0.0001). The 3-year RFS of patients with stage N2 (*n* = 335 [61.2%]) was 18.6% lower (*P* ≤ 0.0001) than the original cohort of patients with stage N1 (*n* = 745 [79.8%]), but it was similar to the RFS of patients with 4 or more tumor deposits plus lymph node metastases (*n* = 58 [61.4%]) (*P* = 0.91). The RFS for patients with 4 or more tumor deposits plus number of lymph node metastases (*n* = 58 [61.4%]) was 15.8% lower than the cohort of patients with 1–3 tumor deposits + number of lymph node metastases (*n* = 687 [77.2%]) (*P* = 0.001). Multivariate analysis confirmed that patients with 4 or more tumor deposits + the number of lymph node metastases (hazard ratio [HR], 1.88; 95% CI, 1.24–2.87) were independently associated with a shorter RFS.

**Conclusion:**

The number of tumor deposits is an indicator of poor postoperative prognosis. It is necessary to incorporate the number of tumor deposits combined with the number of lymph node metastases to stratify postoperative stratification of stage III CRC, which may provide a new theoretical basis for adjuvant therapy for patients with N1 stage CRC after surgery.

## Introduction

Colorectal cancer (CRC) is the third leading cause of cancer death worldwide. [1]. During the standard treatment of CRC, the need for postoperative adjuvant chemotherapy depends on the tumor stage [2–4]. However, tumor node metastasis (TNM) staging cannot provide complete prognostic information of patients. And patients with same tumor stage often have significantly different clinical outcomes [5]. Therefore, it is urgent to develop a new prognostic tool to evaluate recurrence risk of CRC patients, thus personalizing the treatments for patients at high risk of recurrence in advance.

Over the past decade, the location of tumor deposits in the TNM staging of CRC has been changing. Since the 7th edition of AJCC/TNM staging [6], the N staging of CRC introduced the definition of N1c: no regional lymph node metastases but tumor nodules in subserosal, intramesenteric, or nonperitoneal-covered colon/rectal tissues [2–4]. However, the staging method has significant drawbacks. First, more than 80% patients have concurrent lymph node metastases. According to the definition of N1c, once there is lymph node metastasis, the number of tumor deposits does not contribute to staging, which will lead to the patients with only one lymph node metastasis and multiple tumor deposits are still classified as N1a. Second, the number of tumor deposits also affects the patient’s prognosis. Patients with one tumor deposit and patients with multiple tumor deposits have different recurrence risks. To investigate the prognostic role of tumor deposits in stage III CRC, Cohen R et al. performed a retrospective analysis of the GALGB/SWOG 80,702 phase III clinical trial, which revealed that the number of tumor deposits correlated linearly with poor prognosis. And the prognosis of patients newly classified as N2 was significantly worse than that of patients newly classified as N1, where the number of tumor deposits has been added to the number of positive lymph nodes in the newly classifying N1 or N2 [7]. Research results demonstrated the limitations of current N1c staging methods.

Although the clinical trial has proved that the inclusion of tumor deposit numbers in positive lymph nodes is a scientific and convenient staging method, its applicability in the Chinese population remains to be explored. The aim of this study was to evaluate the prognostic value of a new N stage combining tumor deposits (TD) with the number of lymph node metastases in predicting recurrence-free survival (RFS) in stage III colorectal cancer.

## Methods

### Ethics

This multi-center retrospective study was approved by the ethics committee of the Yunnan Cancer Hospital (KY2019141) and the ethics committee of the Sixth Affiliated Hospital, Sun Yat-sen University (2021ZSLYEC-051). The requirement for informed consent was waived by the ethics committee, owing to the study’s retrospective nature. All the patient data in the survey were anonymized.

### Patients

**Fig. 1:**
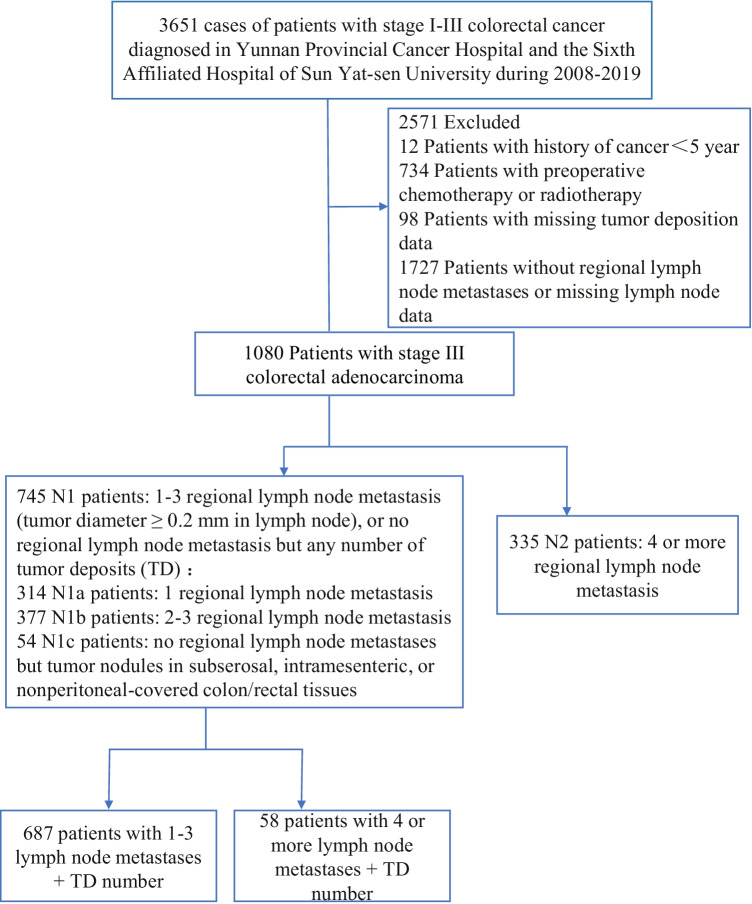
Study flow chart

We retrospectively enrolled 1248 stage III CRC patients who underwent direct surgical resection between April 2008 and February 2019 at Yunnan Cancer Hospital or the Sixth Affiliated Hospital of Sun Yat-Sen University, China. The study flow chart, detail inclusion, and exclusion criteria are shown in Fig. 1.

### Inclusion and exclusion criteria

A total of 3651 patients with stage I–III CRC diagnosed in Yunnan Cancer Hospital and the Sixth Affiliated Hospital of Sun Yat-Sen University from 2008 to 2019 were retrospectively and continuously collected. We excluded 2571 patients with following criteria: [1] patients with cancer history < 5 years (*n* = 12); [2] patients with preoperative chemotherapy or radiotherapy (*n* = 734); [3] patients with missing tumor deposition data (*n* = 98); [4] patients with no regional lymph node metastases or missing nodal data (*n* = 1727).

### Surveillance protocol

All pathology reports were cross-diagnosed by 2 senior pathologists and reviewed by a third pathologist. The number of tumor deposits and lymph node metastasis was recorded in detail. Clinical evaluation included physical examination, serum carcinoembryonic antigen (CEA), carbohydrate antigen 199 (CA199) levels, imaging studies (including contrast-enhanced computed tomography of the chest, abdomen, and pelvis), and colonoscopy. Imaging tests were performed at least every 12 months for more than 3 years. Colonoscopy was done 1 year after surgery and then every 2 to 5 years. All recurrence cases were confirmed by histology or imaging.

### Adjuvant chemotherapy protocol partial

Patients with stage III CRC received the adjuvant chemotherapy according to the National Comprehensive Cancer Network (NCCN) clinical practice guidelines in the CRC. Adjuvant chemotherapy protocol included FOLFOX, CapeOX, Capecitabine, or 5-FU/leucovorin.

### Exposures

According to the number of tumor deposits plus the number of lymph node metastases, patients with original N1 stage were divided into 2 cohorts: 1–3 tumor deposits + lymph node metastases (TD + LN < 4), and 4 or more tumor deposits + lymph node metastases (TD + LN ≥ 4). And patients with original stage N2: 4 or more regional lymph node metastases (LN ≥ 4).

### Definitions of recurrence-free survival

The recurrence-free survival (RFS) was defined as the time interval from the initial surgery until the first recurrence of CRC, death as a result of any cause, or last follow-up. Patients who survived without recurrence or death before the last follow-up were reviewed. Each enrolled patient was completely followed up for 3 years, while those less than 3 years were not enrolled in the study. In total, 1,080 patients were followed up for more than 3 years and met the enrolment requirements, out of which 351 had recurrence and metastasis.

### Statistical analysis

Continuous variables were compared using the Mann–Whitney *U* test. Categorical variables were compared using the *χ* test. RFS was estimated using the Kaplan–Meier analysis. Differences in RFS were assessed by log-rank test (univariate analysis). Hazard ratios (HR) and 95% confidence intervals (CIs) were estimated with Cox regression models and assessed by Wald’s test. Association with RFS was assessed by multivariate Cox regression. Variables with *P*-values < 0.05 in univariate analysis were included in the final multivariate model. All statistical analyses were performed with R software (version 3.2.4). And *P* values < 0.05 were considered significant.

## Results

Finally, 1080 stage III CRC patients (634 [58.7%] men; median [IQR] age, 60 [50–68] years) who underwent radical surgical resection were included in this study. Among the 1080 patients, 745 patients (68.9%) had stage N1 and 335 patients (31.1%) had stage N2 by incorporating the number of tumor deposits into the number of lymph node metastases. Of the 745 patients with stage N1 disease, 687 patients had 1–3 tumor deposits + lymph node metastases and 58 patients had 4 or more tumor deposits + lymph node metastases (Fig. 1) (Table 1).

**Table 1:** Patient characteristics

Characteristic	All (*N* = 1080)	TD-negative (*n* = 844)	TD-positive (*n* = 236)	*P*-value
Age (years)				0.077
Median (IQR)	60.000 [50.0, 68.0]	60.000 [50.0, 68.0]	61.000 [51.0, 68.0]	
Sex, no. (%) of patients				0.328
Male	634 (58.704)	502 (59.479)	132 (55.932)	
Female	446 (41.296)	342 (40.521)	104 (44.068)	
BMI(kg/m2)				0.169
Median (IQR)	22.53 [20.76, 24.97]	22.50 [20.76, 24.87]	22.83 [20.76, 25.26]	
Surgical approach				0.633
OR	687 (63.611)	540 (63.981)	147 (62.288)	
LR	393 (36.389)	304 (36.019)	89 (37.712)	
Primary site, no. (%) of patients				0.053
Right colon	246 (22.778)	206 (24.408)	40 (16.949)	
Left colon	281 (26.019)	216 (25.592)	65 (27.542)	
Rectum	553 (51.204)	422 (50.000)	131 (55.508)	
Tumor differentiation, no. (%) of patients				0.713
Well	41 (3.796)	34 (4.028)	7 (2.966)	
Moderate	577 (53.426)	444 (52.607)	133 (56.356)	
Poor-undifferentiated	397 (36.759)	315 (37.322)	82 (34.746)	
Unknown	65 (6.019)	51 (6.043)	14 (5.932)	
Mucinous type				0.429
No	997 (92.315)	782 (92.654)	215 (91.102)	
Yes	83 (7.685)	62 (7.346)	21 (8.898)	
T stage, no. (%) of patients				0.080
T1	14 (1.296)	13 (1.540)	1 (0.424)	
T2	107 (9.907)	88 (10.427)	19 (8.051)	
T3	908 (84.074)	709 (84.005)	199 (84.322)	
T4	51 (4.722)	34 (4.028)	17 (7.203)	
N stage, no. (%) of patients				< 0.001
N1a	314 (29.074)	266 (31.517)	48 (20.339)	
N1b	377 (34.907)	305 (36.137)	72 (30.508)	
N1c	54 (5.000)	0 (0.000)	54 (22.881)	
N2a	218 (20.185)	175 (20.735)	43 (18.220)	
N2b	117 (10.833)	98 (11.611)	19 (8.051)	
AJCC 7th ed. stage				0.857
IIIA	94 (8.704)	75 (8.886)	19 (8.051)	
IIIB	851 (78.796)	662 (78.436)	189 (80.085)	
IIIC	135 (12.500)	107 (12.678)	28 (11.864)	
LVI				0.005
Yes	135 (12.500)	93 (11.019)	42 (17.797)	
No	945 (87.500)	751 (88.981)	194 (82.203)	
PNI				0.207
Yes	44 (4.074)	31 (3.673)	13 (5.508)	
No	1036 (95.926)	813 (96.327)	223 (94.492)	
Lymph node yield				< 0.001
≥ 12	876 (81.111)	708 (83.886)	168 (71.186)	
< 12	204 (18.889)	136 (16.114)	68 (28.814)	
Adjuvant chemotherapy, no. (%) of patients				0.312
Yes	834 (77.222)	646 (76.540)	188 (79.661)	
No	246 (22.778)	198 (23.460)	48 (20.339)	
Preoperative CEA, ng/mL				0.750
Mean (SD)	20.320 (157.172)	21.128 (176.676)	17.431 (37.287)	
Preoperative CA199, ng/mL				0.820
Mean (SD)	39.864 (197.658)	40.592 (220.663)	37.260 (68.224)	

Data are median (IQR), mean (SD), or *n* (%)

*BMI* body mass index, *CEA* carcinoembryonic antigen, *LR* laparoscopic resection, *LVI* lymphovascular invasion, *OR* open resection, *PNI* perineural invasion. *TD* tumor deposits

*P* value using Wilcoxon Mann–Whitney test, chi-square test or exact Fisher test depending on whether the variable is continuous or categorical

**Fig. 2:**
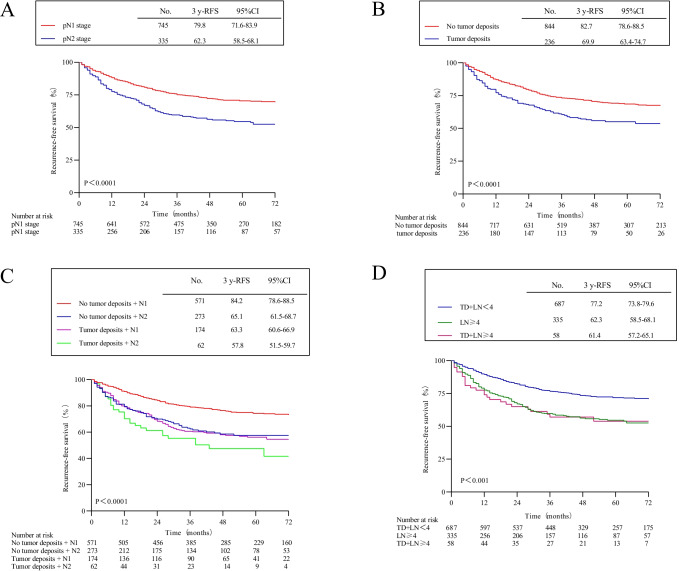
Recurrence-free survival by tumor deposition and pathological N stage. **A** Patients with pN1 stage vs. pN2 stage. **B** Patients with no tumor deposits vs. with tumor deposits. **C** Patients with N1 stage without tumor deposition, N2 stage without tumor deposition, N1 stage with tumor deposition, or N2 stage with tumor deposition. **D** Patients with TD + LN < 4, LN ≥ 4, or TD + LN ≥ 4. (TD, tumor deposits; LN, positive lymph nodes)

The 3-year RFS of 745 patients with original N1 stage was 79.8% (95% CI, 71.6–83.9%), and the 3-year RFS of 335 patients with N2 stage was 62.3% (95% CI, 58.5–68.1%). There was a statistically significant difference between the two groups (HR, 1.82; 95% CI, 1.47–2.26; *P* < 0.0001) (Fig. 2A). The 3-year RFS of 844 patients without tumor deposits was 82.7% (95% CI, 78.6–88.5%), and the 3-year RFS of 236 patients with tumor deposits was 69.9% (95% CI, 63.4–74.7%). And there was a statistically significant difference between the two groups (HR, 1.65; 95% CI, 1.31–2.09; *P* < 0.001) (Fig. 2B). The 3-year RFS of 571 patients with stage N1 without tumor deposition was 84.2% (95% CI, 78.6–88.5%). The 3-year RFS of 273 patients with stage N2 without tumor deposits was 65.1% (95% CI, 61.5–68.7%), which was similar to the 3-year RFS of 174 patients with stage N1 tumor deposits of 63.3% (95% CI, 60.6–66.9%) (N2 patients without tumor deposits vs. N1 patients with tumor deposits: HR, 0.95; 95% CI, 0.71–1.28; *P* = 0.76). The 3-year RFS of the 62 patients with stage N2 tumor deposits was 57.8% (95% CI, 51.5%-59.7%), with the highest risk of recurrence compared with the other three groups (over all log-rank *P* < 0.0001) (Fig. 2C). Among them, the 3-year RFS of 687 patients with tumor deposits and lymph node metastases < 4 was 77.2% (95% CI, 73.8–79.6%), and the 3-year RFS of 335 patients with lymph node metastasis ≥ 4 was 62.3% (95% CI, 58.5–68.1%); the 3-year RFS of 58 patients with tumor deposits and lymph node metastases ≥ 4 was 61.4% (95% CI, 57.2–65.1%) (patients with tumor deposits and lymph node metastases < 4 vs. patients with tumor deposits and ≥ 4 lymph node metastases: HR, 1.40; 95% CI, 1.14–1.73; *P* = 0.001; patients with ≥ 4 lymph node metastases vs. patients with tumor deposits and ≥ 4 lymph node metastases: HR, 1.02; 95% CI, 0.67–1.56; *P* = 0.91) (Fig. 2D).

In the univariate analysis of 3-year RFS, the presence or absence of tumor deposition, lymphatic invasion, perineural invasion, tumor deposition, preoperative CEA, CA199, tumor deposition plus number of lymph node metastases were significantly associated with 3-year RFS (*P* < 0.05). In multivariate analysis, tumor deposition (HR, 1.31; 95% CI, 1.08–2.95; *P* = 0.001), neural invasion (HR, 1.60; 95% CI, 1.17–2.19; *P* = 0.002), preoperative high level of CEA (HR, 1.46; 95% CI, 1.20–1.85; *P* = 0.01), lymph node metastases ≥ 4 patient group (HR, 1.86; 95% CI, 1.49–2.23; *P* = 0.01), tumor deposition plus lymph node metastases ≥ 4 patient group (HR, 1.88; 95% CI, 1.24–2.87; *P* = 0.003) were associated with a shorter 3-year RFS (Table 2). After adjusting confounding factors and incorporating multiple Cox models, the new pathological stage methods consisting of tumor deposits and number of lymph node metastases were independently associated with 3-year RFS (Table 3).

**Table 2:** Univariate and multivariate analyses of 3-year recurrence-free survival

Variables	% 3-year RFS (95% CI)	Univariate		Multivariate	
		HR (95% CI)	*P*-value	HR (95% CI)	*P*-value
Age					
< 60	78.01 (74.16, 82.06)	1.0 (reference)			
≥ 60	77.13 (73.00, 81.50)	1.03 (0.72, 1.09)	0.27		
BMI					
< 24	76.54 (73.02, 80.20)	1.0 (reference)			
≥ 24	79.79 (75.07, 84.80)	0.99 (0.79, 1.23)	0.93		
Sex					
Male	77.95 (74.26, 81.82)	1.0 (reference)			
Female	77.10 (72.74, 81.73)	1.13 (0.91, 1.39)	0.24		
Tumor location					
Right colon	78.01 (74.16, 82.06)	1.0 (reference)			
Left colon	77.13 (73.00, 81.50)	0.97 (0.72, 1.31)	0.86		
Rectum	75.13 (71.29, 79.91)	0.99 (0.76, 1.29)	0.54		
Surgical approach					
OR	76.31 (72.54, 80.28)	1.0 (reference)			
LR	79.39 (75.17, 83.85)	0.93 (0.78, 1.16)	0.52		
Tumor differentiation					
Well	85.71 (77.02, 95.39)	1.0 (reference)			
Moderate	79.05 (75.53, 82.73)	1.23 (0.72, 2.10)	0.43		
Poor-undifferentiated	69.91 (63.77, 76.65)	2.15 (0.98, 3.21)	0.07		
Mucinous type					
No	77.52 (74.63, 80.53)	1.0 (reference)			
Yes	79.62 (66.30, 95.61)	0.81 (0.53, 1.26)	0.76		
Pathology T stage					
T1	92.35 (86.13, 99.03)	1.0 (reference)			
T2	90.14 (85.66, 94.84)	1.61 (0.21, 12.35)	0.64		
T3	73.04 (69.42, 76.85)	5.48 (0.77, 39.07)	0.08		
T4	53.03 (35.38, 79.49)	5.24 (0.69, 39.42)	0.10		
Lymph node yield					
≥ 12	92.35 (86.13, 99.03)	1.0 (reference)			
< 12	90.14 (85.66, 94.84)	0.93 (0.72, 1.21)	0.62		
Adjuvant chemotherapy					
Yes	62.67 (51.41, 76.41)	1.0 (reference)			
No	70.67 (59.71, 75.38)	1.22 (0.93, 1.59)	0.13		
Tumor deposit					
No	82.70 (78.62, 88.54)	1.0 (reference)		1.0 (reference)	
Yes	69.90 (63.43, 74.71)	1.46 (1.18, 1.80)	< 0.001	1.31 (1.08, 2.95)	0.001
Lymphovascular invasion					
No	80.06 (77.17, 83.05)	1.0 (reference)		1.0 (reference)	
Yes	57.33 (47.80, 68.75)	2.45 (1.63, 3.69)	< 0.001	1.50 (0.94, 2.41)	0.08
Perineural invasion					
No	78.87 (76.02, 81.84)	1.0 (reference)		1.0 (reference)	
Yes	54.25 (41.00, 71.78)	1.94 (1.48, 2.54)	< 0.001	1.60 (1.17, 2.19)	0.002
Postoperative CEA, ng/mL					
≤ 5	88.59 (86.48, 91.73)	1.0 (reference)		1.0 (reference)	
> 5	76.65 (69.83, 82.31)	1.46 (1.18, 1.80)	< 0.001	1.49 (1.20, 1.85)	0.01
Postoperative CA19-9, ng/mL					
≤ 37	85.64 (78.92, 89.93)	1.0 (reference)		1.0 (reference)	
> 37	75.44 (70.65, 84.14)	1.52 (1.19, 1.95)	0.001	1.04 (0.91, 2.67)	0.85
New pathology N stage group					
Tumor deposit + lymph node metastases < 4	77.23 (73.81,79.60)	1.0 (reference)		1.0 (reference)	
Lymph node metastases ≥ 4	62.30 (58.49, 68.11)	1.94 (1.56, 2.42)	< 0.001	1.86 (1.49, 2.33)	0.01
Tumor deposit + lymph node metastases ≥ 4	61.42 (57.19, 65.13)	1.98 (1.30, 3.00)	0.001	1.88 (1.24, 2.87)	0.003

*APR* abdominoperineal resection, *BMI* body mass index, *CEA* carcinoembryonic antigen, *LR* laparoscopic resection, *OR* open resection

**Table 3:** Adjusted hazard ratios of 3-year RFS by new pathology N stage group

New pathology N stage group	*N*	Events (%)	Model 1		Model 2		Model 3	
			HR (95% CI)	*P*-value	HR (95% CI)	P Value	HR (95% CI)	*P*-value
Tumor deposit + lymph node metastases < 4	627	93(14.83)	1.0 (Ref.)		1.0 (Ref.)		1.0 (Ref.)	
lymph node metastases ≥ 4	255	61(23.92)	1.94 (1.56, 2.42)	< 0.001	1.89 (1.50, 2.31)	0.009	1.86 (1.49, 2.33)	0.01
Tumor deposit + lymph node metastases ≥ 4	67	36(53.73)	1.98 (1.30, 3.00)	0.001	1.91 (1.28, 2.59)	0.001	1.88 (1.24, 2.87)	0.003

*HR* hazard ratios, *Ref.* reference

Model 1 was unadjusted

Model 2 was adjusted for age (< 60 vs. ≥ 60), body mass index (< 24 vs. ≥ 24), and sex (male vs. female)

Model 3 was adjusted for age (< 60 vs. ≥ 60), body mass index (< 24 vs. ≥ 24), sex (male vs. female), surgical approach (open resection vs. laparoscopic resection), location (right colon vs. left colon vs. rectum), tumor differentiation (well vs. moderate vs. poor-undifferentiated), mucinous type (yes vs. no), pathology T stage (T4 vs. T3 vs. T2 vs. T1), lymphovascular invasion (yes vs. no), perineural invasion (yes vs. no), adjuvant chemotherapy (yes vs. no), lymph node yield (≥ 12 vs. < 12), postoperative CEA, ng/mL (≤ 5 vs. > 5), postoperative CA19-9, ng/mL (≤ 37 vs. > 37), and tumor deposit (yes vs. no)

## Discussion

This study found that patients with stage N1 with tumor deposits had the same risk of recurrence as patients with stage N2 without tumor deposits. Studies have demonstrated that tumor deposition is an independent poor prognostic factor [8]. But in N2 stage, regardless of tumor deposition, survival outcomes are poor [9]. In node-negative patients, tumor deposition was an independent poor prognostic factor for overall survival, disease-free survival, and distant metastasis-free survival. Among node-positive patients, tumor deposition had poor prognostic value only for LN-positive patients [10]. The prognosis of N1c patients is similar to that of node-positive patients without tumor deposits [11]. CRC patients with N1c have a high risk of recurrence and a poor prognosis, and adjuvant chemotherapy should be recommended to improve the prognosis [12]. Some investigators recommend that all node-negative TD patients be included in stage III, regardless of size and shape, should be classified as stage III and should be considered for adjuvant chemotherapy [13]. In rectal cancer, the metastatic risk of tumor deposition is comparable to that of stage pN2, which may lead to changes in adjuvant therapy [14]. The results of a IDEA France phase III study have suggested that the presence of tumor deposition is an independent prognostic factor for disease-free survival in patients with stage III CRC. Adding tumor deposition to lymph nodes may help better define the duration of adjuvant therapy [15].

After restaging by incorporating the number of tumor deposits into the number of lymph node metastases, the risk of recurrence in this subset of patients originally classified as N1 was similar to that of patients newly classified as N2. It was an independent risk factor and was associated with a shorter RFS. Although the prognosis of patients with tumor deposits is poor, the prognosis is better than that of patients with distant metastases [16]. The effect of tumor deposition on overall survival was intermediate between lymph node metastasis and distant metastasis [17]. Tumor deposition alone appears to have prognostic significance similar to lymph node invasion alone [18]. Tumor deposition is associated with the presence of lymph node metastasis and extramural vascular invasion [19]. Tumor deposition and high germination rates are important histopathological variables. It should be used as part of a routine comprehensive pathological risk assessment for stage III colon cancer [20]. The Japanese classification of CRC has described tumor deposits in detail, which determines whether tumor deposits should be considered as metastatic lymph nodes from a prognostic perspective [21]. In patients with lymph node metastases in CRC, staging combined with tumor deposition status may be superior to pN staging in assessing prognosis and survival. It is suggested that tumor deposition status should be included in pN staging [22]. However, the study did not include the number of tumor deposits. Previous study has constructed a prognostic nomogram for patients with stage III CRC, and the number of tumor depositions has a high proportion of prognostic effects [23]. Therefore, it is feasible and scientifically meaningful to incorporate the number of tumor deposits into lymph node metastasis.

A large number of studies have proved the guiding significance of tumor deposition in prognosis. However, some authors suggest that patients classified as T3N2bM0 plus tumor deposition ( +) and T4N2bM0 plus tumor deposition (− / +) should be reclassified as stage IV. In the TNM staging system, the number of tumor depositions is not an independent prognostic parameter [24]. Patients with tumor deposition ( +) stage III CRC have a poor prognosis. And they did not show the DFS benefit from chemotherapy [25].

Our study firstly incorporated the number of tumor deposits into the risk stratification of recurrence and metastasis. Treating tumor deposition as positive lymph nodes in the pN category is feasible and superior to TNM (7th edition) staging [26]. The limitations of this study should be acknowledged and the related areas of research should be further explored. Firstly, the first study was a retrospective analysis. Pathological examination of a large part of the population did not diagnose the number of tumor deposits, and most of the numbers were unknown, which may lead to results offset. Secondly, our study did not include the overall survival analysis. In general, the existing staging can no longer meet the prognosis stratification of patients. It is necessary to incorporate the number of tumor deposits into the comprehensive staging for better risk stratification of recurrence and further help individualize precise treatment.

## Conclusions

After reclassifying the number of tumor deposits to the number of lymph node metastases, patients originally classified as stage N1 had a similar risk of recurrence as patients with stage N2. It is necessary to readjust the current pathological staging of CRC to more accurately predict the prognosis of patients.

## Data Availability

All data and material relevant to the study are available from the authors upon request.
